# Clinical outcomes in TMD patients after arthrocentesis with lysis, lavage and viscossuplementation

**DOI:** 10.1080/07853890.2021.1897446

**Published:** 2021-09-28

**Authors:** J. R. Ferreira, M. A. Nunes, F. Salvado

**Affiliations:** a Universidade de Lisboa, Faculdade de Medicina, Clínica Universitária de Estomatologia. Avenida Professor Egas Moniz, Lisboa, Portugal; b Stomatology and Oral Surgery Department, Centro Hospitalar Universitário Lisboa Norte; c Centro de Investigação Interdisciplinar Egas Moniz (CiiEM), Egas Moniz Cooperativa de Ensino Superior, Caparica, Portugal

## Abstract

**Introduction:**

Temporomandibular disorders (TMD) affect a considerable part of the population [1]. Its most prominent symptoms are restricted joint function with limited maximum mouth opening (MMO), pain and headache, with an important impact on Quality of Life [2,3]. The aim of this study is to evaluate the effect of Arthrocentesis with Lysis, Lavage and Viscosupplementation (ALLV) with hyaluronic acid in the treatment of pain related to TMJ internal derangements.

**Materials and methods:**

Thirty patients diagnosed with internal derangement of the temporomandibular joint through magnetic resonance imaging were submitted to ALLV, after 6 months of ineffective conservative treatment. A classic, single session, two-needle technique arthrocentesis was performed in all cases, encompassing the injection of 200–300 mL of Ringer lactate solution to the superior joint compartment for lysis and lavage, followed by intra-articular injection of 1 mL of hyaluronic acid for viscossuplementation [4,5]. Patients were prescribed a simple program of physical exercises to be repeated daily at home for one month. Evaluation was carried pre-operatively on the day of surgery and post-operatively at 1 week, 1 month, 3 months, 6 months and 12 months after the procedure. Evaluated clinical parameters included: pain (at rest and in function) – Visual Analogic Scale (VAS); maximum mouth opening (MMO) – millimetres (mm); mastication efficiency – VAS. Overall tolerability of the procedure (Likert scale: 0–4) was evaluated at 1-week post-operative time. Data was collected between September 2016 and May 2019. Statistical significance was set at *p* < .05. Paired *t*-tests were used to compare pre- and post-operative pain, MMO and mastication efficiency. Statistical procedures were performed using IBM® SPSS® Statistics – Version 24 and Microsoft® Excel for Mac – Version 16.15.

**Results:**

Twenty-six of the selected patients were women and mean age was 38.86 ± 17.5 years old. In all cases a single side TMJ was treated − 14 left and 16 right. MRI had shown anterior disc displacement (ADD) with complete reduction on opening in 15 patients, without reduction in 9 and with partial reduction in 6. Results show an improvement in pain, both at rest and in function, MMO and mastication efficiency at a statistically significant level, up to 12 months post-operative. The procedure was considered well tolerated (mean Likert: 3.15 ± 1.13) and no adverse events were reported. **Discussion and conclusions:** ALLV is a safe, well tolerated and cost-effective minimally invasive procedure, which proves to reduce pain and functional impairment up to 12 months post-operative, with little or no complications [6,7].
